# C-955-05. Historically Marginalized Burn Survivors: Characteristics Associated with Lost to Follow-Up and Functional Outcomes

**DOI:** 10.1093/jbcr/irag033.154

**Published:** 2026-04-06

**Authors:** Miranda L Yelvington, Kara McMullen, Maxwell B Johnson, Samuel P Mandell, Caitlin M Orton, Jeffrey C Schneider, Huan Deng, Karen J Kowalske, Jennifer Bell-De Paz

**Affiliations:** Arkansas Children's Burn Program, Little Rock, AR; Department of Rehabilitation Medicine, University of Washington, Seattle, WA; Division of Plastic and Reconstructive Surgery, Keck School of Medicine, University of Southern California, Los Angeles, CA; University of Texas Southwestern / Parkland Health, Dallas, TX; Department of Surgery, University of Washington Medicine Regional Burn Center, Harborview Medical Center, Seattle, WA; Spaulding Rehabilitation Hospital, Harvard Medical School, Boston, MA; Spaulding Rehabilitation Hospital, Harvard Medical School, Boston, MA; Department of Physical Medicine and Rehabilitation, University of Texas Southwest Medical Center, Dallas, TX

## Abstract

**Introduction:**

Follow-up after a burn is essential for achieving optimal recovery and has shown a link with an individual’s social determinants of health. A large percentage of burn injuries are sustained by individuals from historically marginalized populations. However, the direct impact of marginalized status on follow-up status and outcomes have not been examined. This study compared loss to follow-up in marginalized populations (MP) compared with a non-marginalized population (NMP), as well as self-reported physical and mental function, and satisfaction with life.

**Methods:**

Data was collected from a national, longitudinal database between 2015-2025 from adults with burn injury. The MP was defined as: age > 65; non-White; Hispanic; Medicaid or public support/indigent; homeless; receiving psychological treatment in the last year or having pre-existing psychological issues; drug or alcohol misuse prior to injury; or pre-existing physical disability. The NMP was characterized by the absence of any of these factors. The primary outcome was follow-up status at 6, 12, and 24 months. Secondary outcomes were last known physical (PCS) and mental (MCS) component summary scores from the VR-12 and the PROMIS Global 10 (scores were cross-walked) and Satisfaction with Life Scale (SWL) scores. Differences between groups were tested univariately using t-tests for continuous varia and chi-square tests for categorical variables. A logistic regression analysis examined the association between follow-up status at 12 months and the demographic characteristics of marginalization in the MP group.

**Results:**

Participants eligible for this study (n = 1608) were divided into MP (n = 1218) and non-NMP (n = 390) groups. Loss to follow-up was not significantly different by marginalized status at either 6 or 12 months. The NMP group had significantly higher PCS (46.8 versus 44.6) and MCS scores at 6 months (53.0 versus 48.9) and in MCS scores at 12 months (52.9 versus 49.5). For the logistic regression model, within the MP group insurance status (OR 1.5), pre-existing physical disability (OR 1.89), and drug use (OR 1.96) all significantly increased the likelihood of LTFU.

**Conclusions:**

While no significant difference existed in LTFU between MP and NMP groups, social and health factors were identified contributing to LTFU in the MP group. When examining functional status, the NMP group had significantly higher scores and better functioning at 6 and 12 months.

**Applicability of Research to Practice:**

Recognition of factors impacting LTFU status can aid in the development of strategies to support those individuals most likely to be lost. Long-term outcomes may be improved through consistent follow-up, which is crucial for guiding individuals to maximum recovery.

**Funding for the study:**

N/A.

